# The association between hydration status and cognitive function among free-living elderly volunteers

**DOI:** 10.1007/s40520-018-1019-5

**Published:** 2018-08-20

**Authors:** Agata Białecka-Dębek, Barbara Pietruszka

**Affiliations:** Department of Human Nutrition, Warsaw University of Life Sciences, Nowoursynowska 159c, 02-776 Warsaw, Poland

**Keywords:** Urine specific gravity, Hydration, Cognitive test, Cognitive function, Elderly

## Abstract

**Background:**

Ageing is inevitably associated with a progressive cognitive decline. With the rising percentage of the elderly in society, the number of people with dementia and cognitive impairment increases. Water is a vital ingredient that must be included in the diet. The impact of hydration status on cognitive performance has been studied only a little so far.

**Aims:**

The objective of the study was to investigate the relation between the hydration status and the cognitive function.

**Methods:**

The study was conducted among 60 free-living volunteers, aged 60–93 years. Data on water consumption were gathered based on 3-day records. The hydration status was assessed in morning urine samples by evaluating urine specific gravity. The cognitive function was tested using the Mini Mental State Examination (MMSE), the Babcock Story Recall Test and the Trail Making Test. Information about depression was gathered by the Geriatric Depression Scale.

**Results:**

The mean daily total water intake was 2441 ± 622 ml, and 70% of respondents met the reference values for an adequate intake. The mean urine specific gravity (1.013 g/cm^3^, range of 1.004–1.025 g/cm^3^) indicated that most of the individuals were in a good hydration state. The average result of MMSE was 27.8, which is connected with mild cognitive impairment. There was no significant relationship between the hydration status and the results of the cognitive function test in the studied population.

**Discussion/conclusion:**

As the elderly volunteers had a good hydration status, there was no significant relationship between cognitive performance and urine specific gravity. It is necessary to replicate the findings of this study with a larger and more diverse sample of older adults.

## Introduction

Water is a major component of the human body, and its amount varies depending on the body composition, i.e. proportion of muscle mass and fat tissue [1]. In the elderly, water accounts for approximately 50% of the body weight. As it is a crucial substance for the metabolism, it plays a role as medium of biochemical reactions, in circulation (blood, lymph), substrate transport across membranes, temperature regulation, fluid-electrolyte turnover, and internal environment homeostasis [2]; even mild dehydration is an undesirable condition.

Dehydration can be a result of pathologic fluid loss, diminished water intake, or both of those reasons occurring simultaneously. The elderly are at a higher risk of dehydration than younger adults because of several changes in the organism during ageing, such as a diminished sensation of thirst, lower ability of urine concentration, and sarcopenia (diminished muscle mass, which causes a smaller fluid reserve). Moreover, polypharmacy is a factor, which can increase fluid loss among the elderly, especially when medicines such as diuretics and laxatives are taken [3, 4]. A limited fluid intake may also be related to a number of other factors, including physical limitations, such as reduced mobility or incontinence, social isolation or dementia, as well as other illnesses [5].

Chronic dehydration causes fluid deficits within cells, which can affect the absorption of medications. Any decrease in the stored body water lowers the plasma volume, which can then decrease the stroke volume and force the body to compensate by raising the heart rate. A lower plasma volume has an impact on sweating and blood flow to the skin and, therefore, impedes thermoregulation [6]. Initial symptoms of dehydration are headaches, fatigue and general malaise. Failure of further consumption of sufficient amounts of water leads to the deterioration of cognitive and neurologic functions, organ failure (renal), and in extreme cases—death [7].

On the other hand, cognitive impairment of the elderly, including dementia, is the reason for a decreased probability to meet self-care needs, including a proper fluid intake [8]. Cognitive functioning in the elderly is associated with many factors, mainly with gender, age and educational level. In addition, lifestyle (smoking, alcohol consumption, physical inactivity) and a lack of social support are also related to decline of cognitive function [9].

A result of dehydration is a worse quality of life; therefore, prevention, early identification and treatment are very important. There is a lot of information on dehydration among hospitalised and institutionalised elderly, but we only have a small amount of information on the situation in seemingly healthy elderly. Hence, the aim of the study was to investigate the relation between the hydration status and the cognitive functions among a chosen group of seemingly healthy elderly.

## Methods

The cross-sectional study was conducted in Warsaw (Poland), among 60 free-living (noninstitutionalised) volunteers, aged 60 years and older. Information about opportunities to participate in the study was distributed by leaflets and was published in the local paper. The study protocol was approved on the 10th of January 2014 by the ethical commission of the National Food and Nutrition Institute in Warsaw, Poland.

Inclusion criteria were age 60 and above; noninstitutionalised; ability to sign an informed consent to participate in the study. Exclusion criteria included diagnosed neurodegenerative disease or depression, or being treated with antidepressants; BMI below 18.5 kg/m^2^; taking corticosteroids, diuretics; vomiting, diarrhoea or fever within the past 3 days; cancer; cardiac failure and acute or chronic renal failure.

### Basic measurements

General information about the respondents was collected with the use of the questionnaire method. The questionnaire contained questions about the socio-economic status, information on the educational level, health status and medical history, selected parameters of lifestyle (smoking, alcohol consumption, dietary supplements usage, self-reported physical activity level), as well as information about feelings of thirst, such as the level of the sense of thirst, dryness in the mouth and refreshed feeling after a drink. The respondents were also asked about the presence of dehydration symptoms (headaches, dizziness, weakness), and situations associated with reducing the amount of fluid intake, such as urinary incontinence, difficulty in using the toilet [10].

Body weight was measured on a calibrated digital scale (Seca 213, Germany) in kg with an accuracy of 0.1 kg. The respondents were asked to stay in light clothing and take their shoes off. Their height was assessed to the nearest 0.1 cm with the use of a portable stadiometer (Seca 2013, Germany). The body mass index (BMI) was calculated according to the equation: BMI = weight (kg)/height (m)^2^. According to WHO [11], a BMI below 18.5 kg/m^2^ means malnutrition, from 18.5 to 24.9 kg/m^2^—normal body weight, from 25.0 to 29.9 kg/m^2^—overweight, and over 30.0 kg/m^2^—obesity.

The blood pressure measurement was done using the standard procedure in a sitting position with the use of an automatic blood pressure monitor (Omron M2 Basic).

### Dietary assessment

Data on food intake were gathered during three non-consecutive days (2 weekdays, and 1 weekend day) with the use of the 3-day food record method. Individuals received detailed written information on how to fill the questionnaire properly. The portion sizes were gathered for beverages in ml/days and for solid foods in g/days. All participants received a digital kitchen scale (with a precision of 1 g). The diet records were complemented with dietitian interviews. The daily water intake and the energy value of the diets were calculated with the use of the Dieta 5. software, which contained a food database by Kunachowicz et al. [12]. The daily water consumption was compared with reference values for an adequate intake (AI = 2000 ml for women and 2500 ml for men) [13].

### Hydration status

The hydration status was tested by measuring urine specific gravity. Each respondent received a plastic container for the urine sample with an individual code. Individuals were asked to bring the urine sample for the next meeting and were informed on how to collect urine (portion of morning urine, a volume of approximately 50 ml). Urine specific gravity was measured using a urometer (Spółdzielnia Rzemieślnicza ‘Żoliborz’, Poland). The urine was poured into a narrow cylinder of 50 ml volume; then, the urometer was placed inside the cylinder. After the cessation of urometer fluctuations, the relative weight of urine was determined to the bottom meniscus. The measurement was performed at a temperature of 20–22 °C. A value of urine specific gravity of less than 1.010 g/cm^3^ may indicate an excessive fluid intake. A value higher than 1.020 g/cm^3^ may indicate mild dehydration [14].

### Cognitive function

The cognitive function was tested using the Mini Mental State Examination (MMSE), the Babcock Story Recall Test and the Trail Making Test (TMT) questionnaires. The Mini Mental State Examination (MMSE) is a 30-point questionnaire test that is commonly used to screen for cognitive impairment. It can be used to help diagnose dementia. The MMSE is a series of simple questions and problems in a number of different mental abilities, including a person’s memory, orientation in time, registration, naming, reading, attention and language. A score of 28 points or above (out of 30) indicates normal cognition, between 24 and 27—MCI (mild cognitive impairment), and below 24 can indicate severe, moderate or mild dementia [15]. The Babcock Story Recall Test is a neuropsychological test of episodic memory. This is a 21-unit story measuring immediate and delayed recall. The total score ranges from 0 to 21. The higher the score, the higher the level of episodic memory [16]. The Trail Making Test is a neuropsychological test of visual search speed, scanning, speed of processing, mental flexibility and executive. There are two parts of the test. Part A is used to examine cognitive processing speed. Test B is used to examine executive functioning. The result is the time to complete the task. The shorter the time, the better the cognitive function [17]. In all subjects, the questionnaire was filled in the same conditions (separate room), at the same time of the day, and by the same trained interviewer. The results were consulted with a psychologist.

### Geriatric Depression Scale—short form

A short version of the GDS (GDS-SF) is a subset of 15 questions from the original GDS-LF with the highest correlation with depressive symptoms. The questions are answered ‘yes’ or ‘no’, and one point is assigned to each answer indicating depression. A score below or equal to 5 points is ‘normal’, a score above 5 points suggests depression, and a result above or equal to 10 points is almost always indicative of depression [18].

### Statistics

The StatSoft Statistica software version 10.0. was used for the descriptive and statistical analyses. The results are reported as means ± standard deviation (SD), as well as range or percentages. A *p* value *≤* 0.05 was considered statistically significant. Differences between groups were tested with the Chi-square test (nonparametric data) or the Student’s *t* test (parametric data). Pearson’s correlation coefficients were used to test the relationship between the cognition function and the daily water intake or hydration status.

## Results

The characteristics of the population under study were studied in relation to sex (Table 1). Of the 60 individuals, 16 (26.7%) were men and 44 (73.3%) were women. The mean age was 70.2 ± 6.6 years. The average body mass index was 27.1 kg/m^2^, and most of the study population was overweight or obese (75% of the study population had a BMI > 25 kg/m^2^, data not given in the table). There were statistically significant differences observed between men and women concerning years of education, marital status, self-assessment of health status, presence of a cardiovascular disease, physical activity level, and dietary supplement usage. Table 2 shows the results of the cognitive function assessment tests and the screening test for depression in the study group. There were no statistically significant differences in the results of all cognitive function tests between genders. The average result of MMSE was 27.8 ± 1.8 points. Taking into account gender, a statistically significant difference was observed in the GDS results; the score was higher for women than for men, and for both groups, the mean value was within the ‘normal’ range (below 5 point). Table 3 shows the characteristics of the total study sample concerning factors connected with the hydration status: daily water intake and urine specific gravity. The mean daily water intake from all sources was 2441 ml, and as many as 70% of respondents met the reference values for an adequate intake (AI). Women were more likely to implement the recommendations at the AI level. Mean urine specific gravity amounted to 1.013 g/cm^3^, which shows that the study population was in a good hydration state. There was a statistically significant difference in the specific gravity of urine depending on gender; men had a higher specific gravity of urine than women (which may be associated with a lower implementation of the recommendations for fluid intake by men).

**Table 1:** Participants’ scores on health, social and lifestyle variables

	Total				Men			Women			*p* value
	M	SD	Range		M	SD	Range	M	SD	Range	*T* test
Socio-demographic characteristics											
Age (years)	70.2	6.6	60–93		71.7	8.0	63–93	69.6	6.0	60–83	0.29142
BMI (kg/m^2^)	27.1	4.2	19.4–43		25.6	3.4	19.4–33.7	27.7	4.3	20.4–43	0.08828
Education (years)	14.3	3.3	7–21		15.9	2.7	11–21	13.7	3.3	7–21	0.01992*

	*n*	%			*n*	%		*n*	%		Chi-square test
Marital status											
Single	24	40.0		3		18.8		21	47.7		
Married	36	60.0		13		81.2		23	52.3		0.04275*
Physical health											
Self-assessment of health status											
Very good	5	8.3			4	25.0		1	2.3		
Good	21	35.0			6	37.5		15	34.1		
Bad	34	56.7			6	37.5		28	63.6		0.01274*
Cardiovascular disease											
Yes	16	26.7			1	6.3		15	34.1		
No	44	73.3			15	93.7		29	65.9		0.03104*
Hypertension											
Yes	25	41.7			6	37.5		19	43.2		
No	35	58.3			10	62.5		25	56.8		0.69301
Diabetes											
Yes	3	5.0			0	0		3	6.8		
No	57	95.0			16	100		41	93.2		0.28390
Lifestyle factors											
Physical activity											
Low	29	48.3			7	43.8		22	50.0		
Moderate	21	35.0			3	18.7		18	40.9		
High	10	16.7			6	37.5		4	9.1		0.02444*
Alcohol intake (current)											
Yes	55	91.7			16	100		39	88.6		
No	5	8.3			0	0		5	11.4		0.15903
Dietary supplement											
Yes	35	58.3			3	18.8		32	72.7		
No	25	41.7			13	81.2		12	27.3		0.00018*
Current smoker											
Yes	6	10.0			1	6.3		5	11.4		
No	54	90.0			15	93.7		39	88.6		0.55930

*M* mean, *SD* standard deviation, *BMI* body mass index

*Statistically significant *p ≤* 0.05

**Table 2:** Results of cognitive assessment tests and screening tests for depression in the study group

Variable/test name	Total			Men			Women			*p* value *T* test
	M	SD	Range	M	SD	Range	M	SD	Range	
MMSE (pt.)	27.8	1.8	22–30	28.1	2.1	23–30	27.7	1.7	22–30	0.50092
Babcock Story Recall (pt.)	9.4	3.1	4–17	10.5	2.7	7–16	9.0	3.1	4–17	0.09454
Delayed recall of Babcock (pt.)	13.4	3.8	6–21	14.2	3.9	8–21	13.1	3.8	6–12	0.33227
TMT part A (sec.)	44.5	14.8	21–98	42.6	14.9	21–78	45.2	14.9	25–98	0.54248
TMT part B (sec.)	109.3	51.6	42–321	111.9	53.4	42–227	108.4	51.5	45–321	0.82675
GDS (pt.)	3.5	2.7	0–12	2.2	2.1	0–8	3.9	2.8	0–12	0.03050*

*M* mean, *SD* standard deviation, *MMSE* Mini Mental State Examination, *GDS* Geriatric Depression Scale, *TMT* Trail Making Test, *pt*. points, *sec*. seconds

*Statistically significant *p ≤* 0.05

**Table 3:** Daily water intake, energy value of the diet, and urine specific gravity in a study sample

Characteristic	Total			Men			Women			*p* value
	M	SD	Range	M	SD	Range	M	SD	Range	*T* Test
Data on water consumption evaluated on a 3-day record										
Daily water intake (ml/day)	2441	622	982–4326	2665	725	1701–4326	2360	567	982–3841	0.09357
Daily calories intake (kcal/day)	1731	442	511–2571	2084	341	1398–2571	1603	405	511–2485	0.00008*

	*n*	%		*n*	%		*n*	%		Chi-square test
Water consumption above the level of the AI										
Yes	42	70.0		8	50.0		34	77.3		
No	18	30.0		8	50.0		10	22.7		0.04149*

	M	SD	Range	M	SD	Range	M	SD	Range	*T* Test
Urine parameters										
Urine specific gravity (g/cm^3^)	1.013	0.005	1.004–1.025	1.016	0.005	1.009–1.024	1.012	0.0044	1.004–1.025	0.00223*

*M* mean, *SD* standard deviation, *AI* reference values for adequate intake (2000 ml for women and 2500 ml for men)

*Statistically significant *p ≤* 0.05

Table 4 shows some additional factors connected with the hydration status: feeling of thirst, dryness in the mouth as well as feeling of refreshment after a drink. In the study group, 28.3% of the respondents reported incidents of incontinence related to activities, such as coughing, laughing or physical strain. Furthermore, 18.3% of the study group had problems with using the toilet and 10% intentionally limited the amount of beverages to reduce the number of times they visited the toilet. The majority of respondents felt thirsty at a moderate level (33.3%) for most of the time, and as much as 23.3% did not feel thirsty at all. More than 80% of the participants did not feel a sensation of dryness in the mouth or felt it for a short period of time. Most respondents felt only slightly refreshed after a drink (33.3%). Table 5 shows the characteristics of the respondents (socio-demographic, physical activity level, dietary supplementation, health status, daily water intake, urine specific gravity) in dependence of the results of MMSE. The study group was divided into two groups taking into account the score of the assessment test—participants with a score of 28 points or more (normal cognitive, *n* = 36) as well as those with less than 28 points (cognitive impairment, *n* = 24). There were no statistically significant differences between these two groups, with one exception—the number of years of education. A statistically, significantly higher percentage of subjects with a MMSE score of 28 points or more (normal cognitive) had a higher number of years of education than those with a score of less than 28 points (58.3% vs. 20.8%, respectively). Correlations between the daily water intake/urine specific gravity as well as the results of cognitive and depression assessment tests are presented in Table 6. We observed no statistically significant relationships between the daily water intake and the hydration status, measured as urine specific gravity and the cognitive function.

**Table 4:** Characteristics of total study sample for factors connected with hydration status

Characteristic	Total		Men		Women		*p* value
	*n*	%	*n*	%	*n*	%	Chi-square test
Feeling dizzy							
Yes	14	23.3	1	6.3	13	29.5	
No	46	76.7	15	93.7	31	70.5	0.05921
Headaches							
Yes	22	36.7	3	18.7	19	43.2	
No	38	63.3	13	81.3	25	56.8	0.08245
Feel weak							
Most of the time	4	6.6	0	0	4	9.1	
Some time	9	15.0	0	0	9	20.5	
Insignificant part of time	31	51.7	10	62.5	21	47.7	
Never	16	26.7	6	37.5	10	22.7	0.10303
Incontinence during the coughing, laughing, physical strain							
Yes	17	28.3	2	12.5	15	34.1	
No	43	71.7	14	87.5	29	65.9	0.10075
Feeling dryness in the mouth							
Most of the time	3	5.0	0	0	3	6.8	
Some time	9	15.0	0	0	9	20.5	
Insignificant part of time	29	48.3	11	68.7	18	40.9	
Never	19	31.7	5	31.3	14	31.8	0.10021
Feeling of thirst							
Not at all	14	23.3	6	37.5	8	18.2	
Very gentle	13	21.7	1	6.3	12	27.3	
Mild	13	21.7	4	25.0	9	20.5	
Average	20	33.3	5	31.2	15	34.0	0.22043
Refreshment after drinking fluids							
Not at all or a little	25	41.7	4	25.0	21	47.7	
Average	16	26.6	5	31.3	11	25.0	
Enough	19	31.7	7	43.7	12	27.3	0.26815
Consciously limiting fluid intake to avoid visiting the toilet							
Yes	6	10.0	0	0	6	13.6	
No	54	90.0	16	100	38	86.4	0.11947

*Statistically significant *p ≤* 0.05

**Table 5:** Differences between group with MMSE score above or below the normal range (MMSE ≥ 28 point), depending on the socio-demographic characteristics, lifestyle and factors connected with hydration status

Characteristic	MMSE score				Chi-square test *p*
	≥ 28, *n* = 36		< 28, *n* = 24		
	*n*	%	*n*	%	
Gender					
Men	11	30.6	5	20.8	
Women	25	69.4	19	79.2	0.40412
Age (years)					
60–70	20	55.6	14	58.3	
71–93	16	44.4	10	41.7	0.83155
BMI (kg/m^2^)					
18.5–24.9	11	30.6	4	16.7	
25.0–29.9	18	50.0	16	66.6	
≥ 30	7	19.4	4	16.7	0.39110
Education (years)					
≤ 14	15	41.7	19	79.2	
> 14	21	58.3	5	20.8	0.00408*
Marital status					
Single	14	38.9	10	41.7	
Married	22	61.1	14	58.3	0.82964
Physical activity					
Low	16	44.4	13	54.2	
Moderate	14	38.9	7	29.2	
High	6	16.7	4	16.6	0.71516
Dietary supplementation (the last month)					
Yes	23	63.9	12	50	
No	13	36.1	12	50	0.28505
Self-assessment of health status					
Very good	3	8.3	2	8.3	
Good	13	36.1	8	33.3	
Bad	20	55.6	14	58.4	0.97465
Daily water intake above AI					
Yes	25	69.4	17	70.8	
No	11	30.6	7	29.2	0.90844
Urine specific gravity					
≤ 1.010 g/cm^3^ (*n* = 21)	10	27.8	11	45.8	
1.011–1.014 g/cm^3^ (*n* = 20)	11	30.5	9	37.5	
>1.014 g/cm^3^ (*n* = 19)	15	41.7	4	16.7	0.11126

*AI* reference values for adequate intake (2000 ml for women and 2500 ml for men), *BMI* body mass index, *MMSE* Mini Mental State Examination

*Statistically significant *p ≤* 0.05

**Table 6:** Correlations between daily water intake as well as urine specific gravity and cognitive and depression assessment tests

Variable/test name	Daily water intake		Urine specific gravity	
	*r*	*p*	*r*	*p*
MMSE (pt.)	0.15777	0.246	0.17034	0.209
Babcock Story Recall (pt.)	0.09455	0.488	− 0.01062	0.938
Delayed Recall of Babcock (pt.)	0.01930	0.888	0.04882	0.721
TMT part A (sec)	− 0.11535	0.397	− 0.19221	0.156
TMT part B (sec.)	− 0.18781	0.166	− 0.16226	0.232
GDS (pt.)	− 0.19743	0.145	− 0.12706	0.351

*MMSE* Mini Mental State Examination, *GDS* Geriatric Depression Scale, *TMT* Trail Making Test, *pt*. points, *sec*. seconds, *r* Pearson correlation coefficient

*Statistically significant *p ≤* 0.05

## Discussion and conclusions

The elderly are considered to be an at-risk population regarding dehydration [5]. Existing evidence is strongly suggestive of high rates of dehydration in the elderly, especially within hospitals and residential care [19]. During hospitalisation, dehydration is independently associated with poorer outcomes, longer length of stay and greater costs [20]. Despite the general consensus that an appropriate level of fluid intake is essential for health [21], little work has been done to date to measure the relationship between fluid intake or the hydration status and the cognitive function. Existing data show that good hydration is connected with better results in cognitive tests, and mild dehydration can impair cognitive abilities [22], but there are only a few studies on the relation of the hydration status and the cognitive performance in elderly. In this study, the relationship between the hydration status and the cognitive performance has not been demonstrated, which is not consistent with the results of previous studies. The study conducted by Suhr et al. [23] on 28 healthy community-dwelling elderly demonstrated that a lower hydration status (defined as the total body water measured by the bioelectrical impedance method) was associated with a decreased psychomotor processing speed, poorer attention and memory. Another study by Suhr et al. [24] conducted on a group of 21 women (mean age of 60 years) showed a relation between the total body water (also measured by the bioelectrical impedance method) and working memory or memory skills. Another study, which examined the effect of age on energy balance, metabolism, hydration and performance during 10 days of strenuous hill walking, showed that the younger group (mean age of 24 years) remained hydrated, whereas the older group became progressively dehydrated (mean age of 56 years). The increased urine osmolality in the older group was correlated with a decline in choice reaction times [25]. It needs to be highlighted that the present study also included seemingly healthy elderly, but the result indicated that the hydration status of individuals was proper; none of the participants were assessed as mild or severely dehydrated. Despite this, men had a statistically significant higher specific gravity of urine than women and less often implemented the recommendations for fluid intake, which is consistent with the literature [26]. Furthermore, a significant percentage of the participants did not feel a sensation of dryness in the mouth or felt it for a short period of time, and for most of time, the majority of the respondents felt thirsty at a moderate level or did not feel thirsty at all.

Some methodological weaknesses of this type of research should be considered, such as different measurements in the cognitive assessment and the hydration state, and a relatively small sample size. Most studies on hydration and the cognitive function are short term and it is not certain if there are longer term cognitive decrements resulting from dehydration. The study by Pross et al. [27] on a group of young, healthy women suggests that even after replenishing a fluid deficit, effects on mood may persist. In view of the fact that the cognitive functioning may still be compromised in young people even after achieving euhydration, the elderly are possibly more exposed to the negative effects of dehydration; however, there is a need for additional research in this area.

It is necessary to replicate the findings of this study with a larger and more diverse sample of older adults. The assessment of hydration is very difficult; the authors of several published review papers claim that a single gold standard for hydration assessment is not possible [2]. In the present study, the hydration status was tested by evaluating urine specific gravity. This method is simple, fast, non-invasive and cheap, so it gives the possibility to use it in large epidemiological studies. It is necessary to develop ways of screening for mild dehydration and recognise the early symptoms leading to dehydration to prevent severe dehydration and its consequences. The relationship between hydration and cognitive performance is an emerging area of study, which requires more research. Future studies should consider an evaluation of water consumption and the state of hydration for longer periods of time to determine whether hydration enhancement leads to improvements in cognitive performance.
